# Arbovirus Prevalence and Vulnerability Assessment Through Entomological Surveillance in Ponce, Puerto Rico

**DOI:** 10.3390/ijerph22060854

**Published:** 2025-05-29

**Authors:** Kayra M. Rosado-Ortiz, Manuel Rivera-Vélez, Ivanna B. Lorenzo-Pérez, Elizabeth M. Ramos-Colón, Mileily Velázquez-Ferrer, Dayaneira Rivera-Alers, Vanessa Rivera-Amill, Robert Rodríguez-González

**Affiliations:** 1 Public Health Program, Ponce Research Institute, Ponce Health Sciences University, Ponce, PR 00716, USA; krosado15@stu.psm.edu (K.M.R.-O.); marivera20@stu.psm.edu (M.R.-V.); ilorenzo24@stu.psm.edu (I.B.L.-P.); eramos@psm.edu (E.M.R.-C.); mvelazquez21@stu.psm.edu (M.V.-F.); dayrivera20@stu.psm.edu (D.R.-A.); 2 Center for Research Resources, RCMI Program, Ponce Research Institute, Ponce Health Sciences University, Ponce, PR 00716, USA; vrivera@psm.edu

**Keywords:** arbovirus, prevalence, vectors, environment, surveillance

## Abstract

The *Aedes aegypti* mosquito is a vector for several arboviral diseases, posing a significant threat to human populations and exacerbating health disparities. Puerto Rico is a subtropical region where *A. aegypti* mosquitoes circulate all the year promoting the transmission of arboviruses. A cross-sectional study in the municipality of Ponce, Puerto Rico was conducted to determine the prevalence of arbovirus in *A. aegypti* mosquitoes and community members, and the impact that sociodemographic and environmental factors on the presence of arbovirus in the community. Our results indicate that more than a third of the population has long-term antibodies (IgG) against chikungunya and the Mayaro virus (56% and 17%, respectively). In addition, more than two-thirds of the population have long-term antibodies (IgG) against dengue and Zika virus (96.0% and 77%, respectively). Dengue virus 1 (DENV-1) was only detected in mosquitoes from urban areas. The practice of storing water in containers uncovered and living near a river increased the odds of having arbovirus in the community (OR = 3.5, 95% CI = 1.8–10.6) (*p* < 0.05) and (OR = 1.6, 95% CI = 1.2–3.7). Furthermore, lower income was a social determinant associated with being at risk of arboviral disease in the communities (OR = 2.9, 95% CI = 1.4–8.5) (*p* < 0.05). It is recommended that public health activities be implemented, including education workshops on prevention and health promotion and health services such as vector control, to prevent arboviral diseases in communities.

## 1. Introduction

Arboviruses are arthropod-borne viruses that have emerged and re-emerged in the Caribbean [1]. Mosquitoes have become the main adversary within arthropods, facilitating the transmission of arboviruses [2]. The *Aedes aegypti* mosquito is a vector that can transmit arboviruses among populations [3]. Furthermore, the *A. aegypti* mosquito has increased its competence, with the potential to spread arboviruses to urban areas in tropical countries [4]. The *A. aegypti* mosquito has led to the transmission of *flaviviruses* (dengue and Zika) and *alphaviruses* (chikungunya and Mayaro), causing a significant impact in the US and the Caribbean islands [1]. An epidemic of chikungunya occurred in 2014, affecting the United States with more than 4659 cases from US territories and 2799 US travelers [5]. In 2016, Zika was leading an epidemic with 36,367 cases in US territories and 4944 US travelers [6]. In 2025, public health authorities from the US declared dengue outbreaks in California, Texas, and Florida and epidemics in Puerto Rico and the US Virgin Islands [7].

The distribution of dengue, chikungunya, and Zika within and across communities, ethnic groups, and different levels of education confirm that social determinants play a role in the presence and spread of these diseases in the Americas [8]. Social determinants of health are the conditions in the societies where people are born, live, learn, work, play, worship, and age that affect a wide range of health in functioning and quality of life [9]. Arboviral diseases are influenced by some of these determinants of health, such as sanitation, infrastructure conditions, education level, and socioeconomic status [10]. Poor sanitation leads to the development of mosquito breeding sites increasing the chances of spreading arboviral diseases [11]. Moreover, unplanned urbanization and lack of adequate households and buildings could also exacerbate the spreading of mosquitoes and arboviral diseases [12]. Related to education, it has been observed that inadequate awareness of arboviral diseases and limited understanding of arbovirus transmission are contributing to the proliferation of arboviruses in developing countries [13]. Furthermore, a lower household income and lack of health insurance are factors that could be related to the increase in arboviral diseases [14].

Human mobility also has gained increasing recognition as a driver of fine-scale dengue risk [15]. By traveling between regions, individuals can unintentionally introduce arboviruses to new areas, where local mosquitoes may become infected and transmit the disease [15]. Population movement increases the chances of contact between infected and susceptible individuals, promoting the spread of arboviral disease [15].

Among other factors, environmental conditions might also be contributing to the increase in mosquitoes and the spread of arboviral disease [16]. Environmental factors influence the distribution and density of *A. aegypti* mosquitoes [17]. Puerto Rico’s warm temperatures, consistent rainfall, and dense population create an ideal environment for the proliferation of *A. aegypti* mosquitoes [17]. Puerto Rico is also affected by storms, floods, and hurricanes annually, which exacerbates the precarious conditions of communities in Puerto Rico and, consequently, increases the risk of arboviral diseases.

Puerto Rico is a subtropical region where mosquitoes circulate year round, promoting the transmission of arboviruses. The *A. aegypti* mosquito is prevalent throughout the island, with higher densities reported in the southern regions of Puerto Rico. In 2024–2025, densities of *A. aegypti* mosquitoes were observed in Ponce, Puerto Rico, using ovitraps. The urban clusters of Punto Oro, Jardines del Caribe, and Baldorioty, showed averages of 112, 86, and 56, respectively. Additionally, higher densities per trap were identified in the rural clusters of Playa, Jaime L. Drew, and Glenview, with averages of 78, 46, and 21, respectively.

Over recent years, epidemics of dengue (2024), chikungunya (2014), and Zika (2016) have affected PR communities, causing high morbidity and mortality rates [9]. Currently, cases of dengue viruses have been confirmed in Puerto Rico, with recent dengue outbreaks contributing to increased morbidity and mortality. The current epidemic of dengue consists of more than 4467 cases, including severe cases and one death [18]. Specifically, Ponce has experienced a surge in dengue cases, with reported numbers rising from 38 to 117, indicating a troubling trend in disease prevalence [18].

The distribution of the vectors, population vulnerability, and their contribution to health disparities at the population level must be studied to prevent and control arboviral diseases in an efficient way. Effective vector-based prevention involves initiating control measures before the beginning of the mosquito season and adult reduction measures following the detection of human arbovirus activity [19]. The aim of this study is to determine the prevalence of arbovirus in *A. aegypti* mosquitoes and community members in Ponce and the impact that sociodemographic and environmental factors have on the presence of arboviruses in the community.

## 2. Materials and Methods

### 2.1. Ethics Statement

This study was conducted per the Declaration of Helsinki, and the protocol was approved by the Institutional Review Board of the Ponce Medical School Foundation, Inc. (IRB approval no: 2305150189). All the participants signed the informed consent document before the sample collection process and completion of the study questionnaires.

### 2.2. Study Participants and Sampling

This study included 100 participants aged 21 years or older, residing in households from clusters identified in Ponce, Puerto Rico. The sample size was determined using as reference values an estimated prevalence of 7%, an acceptable error of 5% (margin error), a confidence interval of 95%, and the total population of Ponce (*N* = 135,674). Participant enrollment was conducted through a sampling process of two stages from July 2023 through September 2023. The first stage consisted of identifying four cluster areas, including two urban areas (Villa del Carmen and Punto Oro), one rural coast area (La Playa), and one rural mountain area (Jaime L. Drew). The second stage was a simple random sampling method for the selection of twenty-five households from each cluster. Households within the cluster areas were numbered, and a list of random numbers was generated using Microsoft Excel. Research assistants then visited the households that corresponded to the numbers on the list. In each household, research assistants proceeded to inform the potential participants about the study’s purpose and protocol. At least one voluntary adult participant was recruited per household by signing the informed consent form. The participants met the following inclusion criteria: currently living in a household from the identified clusters in Ponce, Puerto Rico, and being ≥21 years old. Participants were excluded if they were partial residents in Ponce, Puerto Rico, or if the participant required a legal tutor and it was not available.

Another population under study was mosquitoes collected by ovitraps and vacuums from the identified clusters. Twelve ovitraps were strategically placed in a centralized location, with three ovitraps within each cluster to ensure coverage of all households. Ovitraps were monitored weekly by research assistants. *A. aegypti* mosquitoes were collected, starting in July until October 2023. During the process, bug catcher vacuums were used to collect adult mosquitoes surrounding the ovitraps, paper strips containing eggs were removed from the ovitraps, and larvae and pupae were placed in plastic containers and transported to the laboratory for the completion of the breeding cycle.

### 2.3. Study Design and Data Collection Process

A cross-sectional study was conducted to assess the prevalence of arbovirus in participants and *A. aegypti* mosquitoes from Ponce, Puerto Rico. Research assistants visited 25 households, randomly selected in each cluster area, to present the project, its purposes, and objectives. After presenting the project, the study personnel proceeded to fill out a consent form, allowing each potential participant the opportunity to be part of the study. Household members who decided to participate were identified with a participant’s ID and completed a structured questionnaire that included sociodemographic characteristics, environmental factors, and risk factor variables related to arboviral diseases. Once, the consent form and questionnaire were completed, a phlebotomist (certified research nurse) proceeded to use two tiger tubes of 8.5 mL to take blood samples. After taking the blood samples, each sample was identified with the corresponding participant ID, preserved with ice in a biohazard container, and transported to the molecular laboratory for processing. A Euroimmun serological ELISA test was conducted with the participants’ blood samples to assess the presence of antibodies (IgG and IgM) of DENV, ZIKV, CHIKV, and MAYV obtained due to a previous arboviral infection. Participant’s antibody prevalence was adjusted using the sensitivity and specificity of each serological ELISA test [20].

In the vectors laboratory, samples of mosquitoes collected in the field by ovitraps and vacuums were observed using a Leica stereoscope (v.3.4.7, Leica Microsystems, Durham, NC, USA) and classified by genus and species using entomological guidelines and consulting an entomologist. *A. aegypti* mosquitoes were dissected and preserved in microtubes with RNA-Lock reactive and stored at −80 °C. Pools of ten *A. aegypti* mosquitoes were used for RNA extraction using glass beads and the QIAmp Viral RNA extraction kit (Qiagen, Germantown, MD, USA). The extracted RNA was tested for arboviruses through RT-qPCR assays Roche Lightcycler 480 (Roche Diagnostics, Indianapolis, IN, USA) and CFX96 Bio-Rad. Using RedCap software (version 12.0.8, Bio-Rad Laboratories, Inc., Hercules, CA, USA), all the study information was collected and preserved. To protect the participants’ anonymity, the databases were kept de-identified and were accessible only to study personnel and the principal investigator.

### 2.4. Statistical Analysis

A questionnaire was implemented to obtain data regarding sociodemographic, environmental, and risk factors. Descriptive analysis, including frequencies, proportions, percentages, central tendency measures, and dispersion measures, was completed for sociodemographic characteristics, environmental factors, and risk factors. To determine the prevalence of arboviruses in *A. aegypti* mosquitoes and community members in Ponce, Puerto Rico, descriptive statistics (frequencies and proportions) were used. A table to present frequency and percentage was used to describe the sociodemographic characteristics of the study population. Bar charts were also used to describe geographic and environmental risk factors and the presence of antibodies in participants.

A Chi-square analysis was conducted to assess if there was an association between sociodemographic, environmental, or risk factors and the prevalence of arbovirus in the community. Furthermore, this test was used to measure the association between environmental factors and the prevalence of arbovirus in mosquitoes. Through the association analysis, odds ratios were obtained with their respective confidence intervals and *p*-values.

A logistic regression analysis was conducted to evaluate sociodemographic factors associated with the presence of arboviruses in the community. Also, logistic regression was used to assess if environmental variables were associated with the presence of arboviruses in *A. aegypti* mosquitoes. The Cochran–Mantel–Haenszel method was used to adjust for interactive and confounding variables. Excel (version 16.16.27) was utilized for preparing all study data, and statistical programs SPSS (version 28.0.0.0) and STATA (version 13.0) were used to complete statistical analysis.

## 3. Results

### 3.1. General Characteristics of the Study Participants

A total of 100 study participants were included and analyzed as part of the study, as well as mosquitoes collected from traps in the identified clusters. Table 1 summarizes the sociodemographic characteristics of the study participants.

Results from the study show that the mean age among participants was 57 years (SD = 15.8), and 50.0% of participants were female. Regarding the level of education, 40.0% of participants reported having completed high school, 8% a primary degree, 10% an associate, 11% a professional certificate, 23% a bachelor, 5% a master, and 3% no educational degree. In our study, 21.0% of participants reported having a full-time job, 8% a part-time job, 5% were self-workers, 8% reported being pensioners, 22.0% were retired, 16% stayed at home, 1% were students, 1% were disabled, 10% were unemployed, and 8% did not respond. Almost half of the study sample (47.0%) reported an annual income of less than USD 10,000 annually. A lower income was associated with previous exposition to arboviral diseases (OR = 2.9, 95% CI = 1.4–8.5) (*p* < 0.05). In relation to housing status, 73.0% of participants reported owning a house, 13.0% reported renting, and 14.0% reported living in the house of a family member.

### 3.2. Risk Factors and Arbovirus Prevalence

Table 2 summarizes geographic and environmental factors among study participants. Of the environmental exposure risk factors, 42% of the participants reported living near a body of water, 14% stored rainwater, and 19.0% reported storing water uncovered. Furthermore, 11% of the participants reported having traveled to other countries within the last month.

Table 3 summarizes geographic and environmental factors in cluster areas and the presence of arbovirus. The practice of storing water in uncovered containers increased the odds of having arbovirus in the community (OR = 3.5, 95% CI = 1.8–10.6) (*p* < 0.05). Also, community members whose homes were near a river increased the odds of being at risk of arboviral diseases (OR = 1.6, 95% CI = 1.2–3.7) (*p* < 0.05). Another risk factor, such as travel, yielded no significant associations (*p* > 0.05).

**Figure 1:**
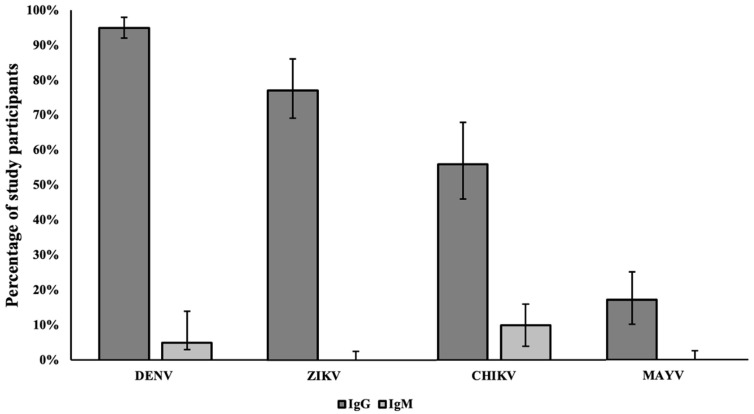
Arboviral antibodies in participants recruited from Ponce, Puerto Rico.

Study results show 96% [95% CI: 93 to 99%] of participants had IgG, and 5% [95% CI: 2 to 9%] had IgM antibodies against DENV. Also, 77% [95% CI: 69 to 86%] of participants had IgG and 0% IgM antibodies against ZIKV. A test to identify alphavirus antibodies (CHIKV or MAYV) was also performed. Results showed that 56% [95% CI: 46 to 68%] of participants had IgG and 10% [95% CI: 4 to 16%] had IgM antibodies against CHIKV, and 17% [95% CI: 10 to 25%] had IgG and 0% had IgM antibodies against MAYV (Figure 1) Although there are no confirmed cases of MAYV in Puerto Rico, some participants presented long-term antibodies against the Mayaro virus due to cross-reactivity with the chikungunya virus. In Figure 1, the upper and lower limits of the 95% confidence interval were included for each percentage of participants.

In each cluster area, three ovitraps were strategically located for a total of twelve ovitraps covering the participant household range and collecting A. aegypti mosquitoes. A total of 335 A. aegypti mosquitoes were collected. The urban cluster of Villa del Carmen had the highest density of A. aegypti mosquitoes, with an average of 49 mosquitoes collected per trap, totaling 148. The second cluster with the highest density of A. aegypti mosquitoes was the rural cluster La Playa, which had an average of 41 A. aegypti mosquitoes collected per trap, totaling 124 mosquitoes. In contrast, the clusters with lower densities of A. aegypti mosquitoes were Jaime L. Drew and Punto Oro, with averages of 12 and 9 mosquitoes per trap, totaling 36 and 27 mosquitoes, respectively.

Of the collected mosquitoes, none tested positive for MAYV, CHIKV, or ZIKV. As shown in Table 4, A. aegypti mosquitoes were found in rural and urban areas alike; however, dengue virus serotype 1 (DENV-1) was only detected in A. aegypti mosquitoes from urban areas, Punto Oro, and Villa del Carmen. From 335 A. aegypti mosquitoes collected, 33 pools (10 mosquitoes per pool) were prepared for molecular testing. Three pools from Villa del Carmen (*n* = 30) and one pool from Punto Oro (*n* = 10) tested positive for DENV-1, resulting in a prevalence of 12% (*n* = 40) among the 335 A. aegypti mosquitoes collected.

## 4. Discussion

In Puerto Rico, arboviruses constantly emerge and re-emerge, causing outbreaks and posing a major public health concern. Our findings provide insight into the prevalence of arbovirus in communities and in *A. aegypti* mosquitoes in Ponce, Puerto Rico. Furthermore, it describes the impact sociodemographic factors and environmental conditions have on the prevalence of arboviruses in the community and the distribution of *A. aegypti* mosquitoes. Sociodemographic, environmental, and risk factors related to arboviral diseases have been assessed recently due to the significant public health burden these viruses impose on communities [14]. Social determinants, such as sanitation, infrastructure conditions, education level, and socioeconomic status, may serve as indicators for the identification of populations that may be more susceptible to arboviral diseases [14].

Therefore, our study assessed sociodemographic and environmental factors. Dalvi and colleagues emphasize the importance of evaluating these factors since research in Brazil has demonstrated their association with the risk of arboviral diseases, such as dengue, chikungunya, and Zika [21]. Following the assessment of these sociodemographic and environmental factors, sero-surveillance was conducted by certified research nurses. Through this process, our study had the purpose of alerting public health authorities in the US and Puerto Rico about the impact of arboviral diseases on the population.

During our assessment of sociodemographic factors, we observed that the majority of the Puerto Rican population within our catchment area consisted of adults, leading to a sample with a mean age of 57 years (SD = 15.8). In terms of sexuality, our sample was evenly distributed, with males and females each representing 50% of the population. Compared to a vector control study conducted in Ponce, Puerto Rico, during 2018–2019, our participants’ characteristics differed slightly from those reported by Sanchez, who found a mean age of 37 years and that 63% of participants were female [22]. The increase in the age of the population observed in our study may result from young people emigrating to the US and the aging population remaining in Puerto Rico.

The demographic profile of our study population revealed that 40% of participants had attained a high school level and 8% had completed only primary education. These data reflect that a high proportion of participants (48%) did not pursue any higher education after high school or primary school. This high percentage of the population (48%) indicates that many community members may face difficulties in understanding arboviral diseases and how they are transmitted. Similar educational levels were reported by Adams in a study conducted in Puerto Rico during 2018–2019, where most of their participants (57.4%) had only a high school degree and no further educational level [23]. Regarding socioeconomic status, 47% of our participants reported an annual income below USD 10,000; which is considered below the poverty level. Participants living below the poverty level were identified among all clusters, although most of them were residents from the rural clusters. Adams and colleagues, in their cohort study, also found a high proportion of participants (44%) living with an annual income below the poverty level (USD 10,000) [23].

The occupational distribution within our study population revealed that only 21% of our participants were employed full time. Of the remaining 79% of the population, 8% had a part-time job, 5% were self-workers, 8% were pensioners, 22% were retired, and 36% were non-working participants. This is an indicator that most of the participants (79%) may not have the economic stability to afford repellents, insecticides, and larvicides, among other resources required to effectively reduce the density of mosquitoes and prevent arboviral diseases. A study by Eldigail and colleagues indicates that populations with lower socioeconomic status, particularly those experiencing unemployment, may have a higher prevalence of arboviral diseases like dengue, suggesting an association between socioeconomic factors and the risk of arboviral diseases [24].

A previous study conducted in Puerto Rico in 2023 showed that CHIKV and ZIKV infection were associated with multiple factors, including older age, lower education level, employment type, public insurance, and lower household income [23]. In our analysis, participants with lower incomes had 2.9 times higher odds of being infected by an arbovirus, and this association was statistically significant (95% CI: 1.4–8.5, *p* < 0.05). In recent decades, Puerto Rico has undergone a dramatic population composition change and economic downturn [25], as seen in the 47% of the population of our study who have an income of less than USD 10,000 annually. As a result, more families are living in poverty levels and have fewer resources, which can affect the ability of communities to have access or be receptive to preventive measures from public health dangers such as vector-borne diseases.

Sociodemographic factors are not the only contributors to vector density and the prevalence of arboviral diseases. Geographic and environmental factors influence the abundance and distribution of *A. aegypti* mosquitoes and the transmission of arboviruses [26]. Multiple factors related to the environment, demographics, and socioeconomic status contribute to the spread of arboviral diseases [26]. Among these factors are human behavior, socioeconomic status, and environmental parameters (temperatures, precipitation, and others) [26]. In our study, we explored geographic and environmental factors. It was observed that participants whose homes were near a river were 1.6 times more likely to be at risk of arboviral diseases. This factor contributed to a suitable environment for mosquito breeding sites and a higher density of mosquitoes. Furthermore, those who had uncovered water storage were 3.5 times more at risk of being infected by the *A. aegypti* mosquito. In Puerto Rico, people often store water, which has posed a challenge in reducing arboviral diseases over the last decade. However, through targeted interventions, the population can be educated on how to cover water containers and thereby prevent mosquito breeding sites. Talbot also emphasizes that water containers can contribute to a higher density of mosquitoes and the spread of arboviral infections [27]. However, using covers on these containers can serve as an effective preventive measure to reduce the risk of creating mosquito breeding sites [27].

In addition to examining sociodemographic and environmental factors, an entomological assessment was conducted to monitor the *A. aegypti* mosquito and the prevalence of arboviruses in communities from Ponce. A total of 335 *A. aegypti* mosquitoes were collected from four different clusters in Ponce and assessed for CHIKV, MAYV, ZIKV, and the four serotypes of DENV through RNA extraction (Qiagen kit) and RT-qPCR assays. Through the assessment, only DENV-1 was found in the collected *A. aegypti* mosquitoes. Moreover, DENV-1 was prevalent in 12% (*n* = 40) of the *A. aegypti* sample (*n* = 335). The entomological assessment was shown to be useful in identifying areas that were at higher risk of arboviral diseases. In our study, those areas were Villa del Carmen and Punto Oro, both urban areas with concurrent movement of human population. Similarly to our results, a Latin American study detected ten positive DENV pools from Nicaragua and Ecuador, including one pool from an urban region [28]. However, our results were higher than those reported in a study from an urban area in Colombia, which found that 3.0% of *A. aegypti* pools were positive for DENV-1 [29]. These findings show that entomological surveillance of mosquitoes serves as an additional tool that aids in detecting arboviral diseases that may be present in the communities before people start becoming infected, which helps to be more effective in preventing arboviral disease.

Epidemiological data from Puerto Rico between 2010 and 2024 indicate a substantial burden of dengue cases, reporting a total of 39,094 cases [30]. A localized study in Ponce from May 2018 to June 2019 revealed a 39% arboviral seroprevalence among 1845 participants [31]. Consistent with these prior observations of arboviral exposure in Puerto Rico, our serological analyses demonstrated widespread antibody presence among our study participants, indicating prior exposure to arboviruses. The study showed that 96% of participants exhibited IgG antibodies against dengue virus (DENV), with 5% showing IgM antibodies, suggesting a mix of past and recent infections. For Zika virus (ZIKV), 77% of participants presented IgG antibodies. Regarding chikungunya virus (CHIKV), 56% of participants displayed IgG antibodies, and 10% exhibited IgM antibodies. Finally, Mayaro virus (MAYV) seroprevalence was also observed, with 17% of participants showing IgG antibodies. Because of the endemic nature of arboviruses in Puerto Rico, these findings are consistent with the prevalence of arboviral diseases in Puerto Rico. These results are useful in providing scientific insights regarding arboviral diseases and contribute relevant data to the public health authorities of Puerto Rico. Future studies are encouraged to combine vector monitoring with community monitoring to provide a more comprehensive understanding of the prevalence of arboviruses and arboviral diseases in the population.

## 5. Conclusions

Arbovirus in Puerto Rico is a continuous public health problem affecting most communities. As public health professionals, it is important to promote strategies and resources that help monitor vectors and identify the prevalence of arboviruses in Puerto Rico. This study overviews the importance of studying underlying sociodemographic and environmental factors and their impact on the presence of arbovirus in the community. There are many risk factors that contribute to a higher frequency of mosquitoes and the prevalence of arboviruses in cluster areas. Communities should take care of their surroundings by removing debris and standing water, and they should avoid storing water to prevent mosquito breeding sites. Regarding having animals at home, in the case of pets, their designated areas must remain clean. For farm animals, keep a safe distance between home and livestock, and ensure the livestock area is clean.

Public health outreach activities that include education, prevention, promotion, and health services are recommended to prevent arboviral diseases in Puerto Rico. The findings from our study showed that certain factors, such as lower educational level, low income, and environmental factors, increase the odds of arbovirus in these communities. The most effective way to maintain a healthy population is by empowering them with knowledge and resources. For that reason, in future studies, the implementation of educational workshops and activities for vector control is recommended, involving community members to encourage commitment to preventing arboviral diseases.

## Figures and Tables

**Table 1:** Demographic characteristics of study participants, Ponce, Puerto Rico, July–December 2023.

Participant Characteristics	(*N*) 100
Sex	
Male	50
Female	50
Age	
21 to 29	9
30 to 39	5
40 to 49	14
50 to 59	19
60 to 69	26
70 to 79	20
80 years or more	7
Level of education	
Primary	8
High school	40
Associate degree	10
Professional certificate	11
Bachelors	23
Masters	5
None	3
Occupation	
Housewife	16
Unemployed	10
Part-time job	8
Full-time job	21
Self-worker	5
Student	1
Pensioner	8
Retired	22
Disabled	1
None/No response	8
People per household	
One to three persons	81
Four to six persons	19
Annual income	
Less than USD 10,000	47
USD 10,000–USD 20,000	26
USD 20,001–USD 30,000	13
USD 30,001–USD 40,000	4
USD 40,001–USD 50,000	6
USD 50,001–USD 60,000	2
More than USD 60,000	2
Housing status	
Rent	13
Own	73
House of a family member	14

**Table 2:** Geographic and environmental factors among study participants, Ponce, Puerto Rico, July–December 2023.

Risk Factors	(N) 100
Store rainwater	14
Store water uncovered	19
Live near a body of water	14
Travel to other countries in the last month	11

**Table 3:** Geographic and environmental factors in cluster areas and the presence of arboviruses (flavivirus and alphavirus) transmitted by *A. aegypti* mosquito, 2023.

Risk Factors	aOR	(95% CI)
Store rainwater	2.1	(0.7–6.15)
Store water uncovered	3.5	(1.8–10.6)
Lives near a body of water	1.6	(1.20–3.7)
Travel to other countries in the last month	1.2	(0.32–1.4)

Note: ORs were estimated by logistic regression after adjusting for age and sex.

**Table 4:** Distribution of A. aegypti mosquitoes and arboviral detection, 2023.

ID	Cluster Areas	*A. aegypti* (*f*)	Arbovirus Detected
U1	Punto Oro	27	DENV-1
U2	Villa del Carmen	148	DENV-1
R1	Jaime L Drew	36	-
R2	La Playa	124	-

## Data Availability

The original contributions presented in this study are included in the article. Further inquiries can be directed to the corresponding authors.
